# Vascular TOS—Creating a Protocol and Sticking to It

**DOI:** 10.3390/diagnostics7020034

**Published:** 2017-06-10

**Authors:** Meena Archie, David Rigberg

**Affiliations:** Division of Vascular Surgery, Department of Surgery, Ronald Reagan Medical Center at the University of California, Los Angeles, CA 90095, USA

**Keywords:** thoracic outlet syndrome (TOS), thoracic outlet syndrome, vascular TOS (VTOS), arterial TOS (ATOS)

## Abstract

Thoracic Outlet Syndrome (TOS) describes a set of disorders that arise from compression of the neurovascular structures that exit the thorax and enter the upper extremity. This can present as one of three subtypes: neurogenic, venous, or arterial. The objective of this section is to outline our current practice at a single, high-volume institution for venous and arterial TOS. VTOS: Patients who present within two weeks of acute deep vein thrombosis (DVT) are treated with anticoagulation, venography, and thrombolysis. Those who present later are treated with a transaxillary first rib resection, then a two-week post-operative venoplasty. All patients are anticoagulated for 2 weeks after the post-operative venogram. Those with recurrent thrombosis or residual subclavian vein stenosis undergo repeat thrombolysis or venoplasty, respectively. ATOS: In patients with acute limb ischemia, we proceed with thrombolysis or open thrombectomy if there is evidence of prolonged ischemia. We then perform a staged transaxillary first rib resection followed by reconstruction of the subclavian artery. Patients who present with claudication undergo routine arterial duplex and CT angiogram to determine the pathology of the subclavian artery. They then undergo decompression and subclavian artery repair in a similar staged manner.

## 1. Introduction

Thoracic Outlet Syndrome (TOS) is a general term used to describe various disorders that arise from compression of neurological or vascular structures that exit the thorax to enter the upper extremity. The thoracic outlet is comprised of the narrow aperture created by the first rib, surrounding musculature and the clavicle. Together, these structures surround the subclavian vein, artery and brachial plexus as they travel distally to the arm. These disorders, as a whole, are rare. The three main forms in decreasing prevalence are neurogenic, venous (also known as Paget Schroetter Syndrome) and arterial. Though uncommon, this disorder is one that all vascular surgeons will encounter at some point, and it is imperative to not only be able to diagnose but also manage these patients.

Doctors have long recognized the occurrence of compressive symptoms due to the anatomical constraints at the thoracic outlet. Historical literature illustrates that this association dates back to Galen’s study of cervical ribs. In the early 19th century, Sir Astley Cooper studied the thoracic outlet in relation to subclavian artery aneurysms. Gruber, Coote, Mayo, Halsted, Paget, and Schroetter are just a few additional names on the long list of historical anatomists and surgeons who recognized the compression of the thoracic outlet and its effects on the neighboring neurovasculature. In 1956, Peet coined the term TOS, and the initial first rib resection was performed in 1962 by Clagett. The transaxillary approach can be attributed to Roos, who was inspired by the transaxillary sympathectomy.

It is estimated that 5000 patients are affected by TOS per year worldwide and 3000 operations are performed annually for this condition. The anatomy of the thoracic outlet predisposes the body to this syndrome given the numerous vital structures that traverse a relatively small aperture. The thoracic outlet is comprised of the area created by the edge of the first rib inferiorly, the clavicle and subclavius muscle superiorly and anteriorly, and the anterior and middle scalenes laterally and posteriorly. The subclavian vein is first to exit from an anterior perspective, just lateral to the subclavius muscle. The anterior scalene then inserts into the first rib and sits between the subclavian vein and artery. Next come the subclavian artery and brachial plexus. Finally, the middle scalene muscle is typically the final compressive structure in the thoracic outlet [1].

Based on the subtype of TOS, variations of pathology exist. Venous TOS commonly presents with hypertrophied anterior scalene and subclavius muscles, as patients are typically younger and more muscular. Arterial TOS is typically seen in patients with bony prominences or by additional structures such as a cervical rib [2].

There are several less common anomalies that have been discovered, and the most thorough classification system that exists is likely that of Roos. In this system, there are ten anatomical variations that lead to TOS, which include cervical ribs, additional or prominent tendons, and additional accessory muscles such as the scalenus minimus.

## 2. Diagnosis of Paget-Schroetter Syndrome

Venous TOS, or Paget-Schroetter Syndrome, typically presents as a sudden-onset phenomenon in an otherwise healthy patient. The typical patient is young, athletic, and might even develop symptoms after a rigorous work out leading to the term “effort thrombosis”. Examples include weight lifters, swimmers, volleyball players, and baseball players. The right side is affected in 60–80% of patients [3].

Clinical evaluation for the Paget-Schroetter patient begins with history and physical examination. The history is quite consistent—a healthy, young patient with complaints of sudden swelling of the entire upper extremity. Discomfort, heaviness, and cyanosis are not uncommon. Patients are typically between the ages of 14 and 45, and are usually involved in a work or leisure-related activity with repetitive movements overhead. It is important to note that venous TOS may occur in both athletes and non-athletes. Some studies show that males are affected at a rate of 2:1 versus women [3]. On physical examination, the affected arm is edematous and sometimes cyanotic. Patients often have obvious collateral veins across their shoulder, neck, or chest. The most common presentations are visible collateral veins across the shoulder (99%), upper extremity edema (96%), bluish discoloration (94%), and aching pain with exertion (33%) [4,5].

It is important to develop an efficacious pathway in the management of TOS given the rarity of the disease. In the following sections, we will outline an algorithm that we have found useful in effectively treating TOS.

Once the diagnosis is entertained, we perform a venous duplex. Given the location of the thrombosis, it may be difficult for the ultrasonographer to demonstrate a DVT. Overall, however, this test is highly sensitive and specific in the diagnosis of axillo-subclavian DVT, with sensitivity approaching 97% and specificity approaching 96% [6]. A negative study does not rule out vascular TOS (VTOS) despite these high values. It is important to note that upper extremity DVTs place the patient at risk for pulmonary embolism. However, due to the mechanical compression, it is uncommon that Paget-Schroetter patients suffer from clinically significant pulmonary emboli [7]. Following a positive duplex scan, or if the clinical suspicion is high, we initiate anti-coagulation. If there is an unavoidable delay before imaging can be performed, we will start anticoagulation before the diagnostic work up is completed.

Once the upper extremity DVT is diagnosed and anticoagulation is begun, we proceed to definitive imaging; the vast majority of patients undergo catheter-based venography. However, the diagnosis can also be confirmed with CT or MR venography. When possible, we perform the initial diagnostic venogram with the intention of treating.

## 3. Management of Paget-Schroetter Syndrome

Following the confirmation of the diagnosis with contrast-enhanced imaging, the patient is a candidate for thrombolysis, especially if the diagnosis has been made within 3 weeks. Access is typically achieved by ultrasound guidance of the ipsilateral basilic vein. Our preferred method is to access the basilic vein with a 4F micropuncture needle, then upsize to a short 5F sheath. Thrombolysis is typically achieved by catheter placement (i.e., McNamara infusion catheter, Covidien, Ireland) in the subclavian vein and infusion of a thrombolytic agent, such as tissue plasminogen activator (Alteplase, Genentech, CA, USA). Our preference is to run the tPA at 0.5 mg/kg/h, though this should be titrated both to clot burden on venography and to serial fibrinogen levels. We maintain the fibrinogen level above 200 mg/dL or >50% of the patient’s pre-operative level. Heparin is also infused at a rate of 400 units/h through the sheath to maintain patency. Serial partial thromboplastin times are drawn every six hours to avoid supra-therapeutic heparin infusion. Thrombolysis typically lasts for 48 to 72 h with lysis checks every 24 h. Once patency has been achieved, lysis is completed and the sheath is removed from the basilic vein. The patient is then placed on therapeutic anticoagulation.

It is important to note that not all groups proceed with thrombolysis prior to surgical decompression. A recent study demonstrated that preoperative thrombolysis did not provide benefit compared to simple anticoagulation [8]. The retrospective study analyzed 110 patients who suffered from acute subclavian vein thrombosis. Forty-five of these patients underwent thrombolysis and sixty-five were treated with anticoagulation alone. In both groups, 91% were ultimately patent with symptom improvement. Since there was no significant difference between the two groups, the efficacy of thrombolysis was brought into question and further investigation is warranted. However, this study focused on subacute and chronic presentations of VTOS. Clinical acumen must be exercised, as thrombolysis has shown benefit in treatment of acute subclavian vein thrombosis in VTOS patients.

Once patency of the subclavian vein is established, consideration is given to resection of the first rib. It is now widely accepted that anticoagulation alone is not sufficient for the treatment of venous TOS [9,10]. Historically, successful thrombolysis was followed by three months of therapeutic anticoagulation, after which a trans-axillary first rib resection and scalenectomy were performed as originally described by Kunkel and Machleder [7]. This three-month period from the time of thrombolysis to the first rib resection may increase the risk of re-thrombosis [1,10,11]. The current standard of care is to minimize this waiting period; many even advocate immediate decompression during the same hospitalization [1,10]. It is our current practice to discuss the timing with the patient. We frequently perform rib resection during the initial hospitalization, but can certainly delay operation if the patient has a need to delay it.

Following first rib resection, it is common to have residual stenosis of the subclavian vein due to fibrous strictures or post-thrombotic changes. Reviews of post-operative venograms indicate that approximately 30–45% of patients have such residual lesions [11]. Our preference is to image patients with catheter-based venography and intra-vascular ultrasound (IVUS) two weeks following surgical decompression. In our experience, IVUS provides three-dimensional imaging of the degree of stenosis, which aids in decision-making. We continue anti-coagulation from the postoperative period until we perform these studies. If we find residual venous disease, we perform a venoplasty at the time. Our experience shows that the majority of lysed veins remain patent on post-operative venography, though this has not been studied. If no lesions are found at the 2-week postoperative venogram, we discontinue anticoagulation. If there is residual stenosis, we continue anticoagulation for 2–4 weeks. At this point, we repeat imaging. Only after we have demonstrated a healthy appearing vein 2–4 weeks following an intervention do we stop the anticoagulation. If we are unable to achieve this result after repeated attempts, we will discuss with the patient with the recommendation to continue anticoagulation for up to 12 months to allow for possible improvement in the vein, including the possibility of late recanalization [3].

There is also a sub-group of patients who will thrombose their axillo-subclavian vein between the time of first rib resection and follow up venography. It is essential to consider incomplete decompression as the etiology. Our approach to these patients is to repeat lysis as necessary after the rib has been removed. Venography with IVUS during the lysis completion procedure should reveal a decompressed thoracic outlet. If this is not the case, the patient should be considered for reoperation [12]. At this point, the patients follow the algorithm for post rib resection vein management. (Algorithm A1). Finally, there is a group of patients who experience repeat thrombosis of their axillo-subclavian vein in the interval from the original lysis to rib resection. This occurs in about 34 percent of patients, though the number is thought to have decreased with the increased utilization of lysis and rib resection at the same hospitalization [13,14]. We proceed with rib resection in these patients, and then perform repeated lysis as necessary. Again, the patients are then on our algorithm for the post-rib excision vein management as above.

Although post- first rib resection subclavian vein stenting remains the subject of ongoing review, we avoid the use of stents in this location. It is important to note that this is in reference to post-surgical stenting as opposed to subclavian vein stenting in the non-decompressed thoracic outlet, which has been widely rejected [4,5].

## 4. Management of Chronic Subclavian Vein Thrombosis in Paget-Schroetter Syndrome

Although there is no definitive length of time for chronic versus acute subclavian vein thrombosis, many authors define acute as less than two weeks and chronic as greater than two weeks [15]. In our experience, surgical decompression takes precedence in the management of patients with symptoms that have been present greater than 3 weeks. We initiate therapeutic anticoagulation in the outpatient setting. Trans-axillary first rib resection is then performed, usually within 2–4 weeks of starting anticoagulation. Two weeks post-operatively, a venogram is performed along with any required intervention including mechanical, on-table, or extended thrombolysis [16,17]. Mechanical thrombolysis yields mixed results, but may be beneficial about 50% of the time [8]. There is not much data regarding on-table lysis; however, we opt to use this technique in patients with residual thrombosis after decompression if the thrombus is relatively small in appearance and the residual stenosis is minimal. If a more significant thrombus burden is seen, extended lysis is performed as described above. It is also accepted that the subclavian vein may recanalize after decompression alone, even if it remains occluded following surgical decompression [17]. This is further reason for decompressing patients who have had a more long-standing occlusion of the axillo-subclavian vein. Again, our protocol is to continue anti-coagulation until a patent, healthy-appearing vein is demonstrated on follow up imaging—typically two weeks after the last intervention. If this cannot be attained, we continue anticoagulation for several months to encourage spontaneous clot resolution.

## 5. Contralateral Asymptomatic Lesions

A small subgroup of patients will present with unilateral symptoms despite significant compression of the contralateral side on imaging. We favor treating a very small minority of these patients with decompression prophylactically, though we recognize this is controversial and is not supported by the literature. Transaxillary first rib resection is our preferred method in these patients as well.

## 6. Venous Reconstruction and Other Surgical Techniques

A small subgroup of Paget-Schroetter patients will require further surgery after venoplasty, thrombolysis, and conventional surgical decompression fail to correct their symptoms. It is important to stress that these procedures should only be considered in patients with persistent and disabling symptoms from an occluded axillo-subclavian vein. The final end point should be symptom status, not necessarily radiologic patency of the subclavian vein. In the rare cases where open venous reconstruction is needed, we have used an infraclavicular approach with placement of an interposition graft. It is essential to ensure that the venous inflow from the brachial vein is adequate to maintain patency of the repair. The best choice of graft is native vein, such as the saphenous. The patient is typically maintained on 3–6 months of anticoagulation afterwards [18].

## 7. VTOS Case Presentation

An otherwise healthy 36 year-old female athlete presented to an outside hospital with an acutely swollen right arm. Acute right axillosubclavian vein thrombosis was diagnosed by a combination of duplex ultrasound imaging and venography. She underwent thrombolysis at that hospital, then presented to us for further management. She had been anticoagulated with Rivaroxaban.

**Figure 1:**
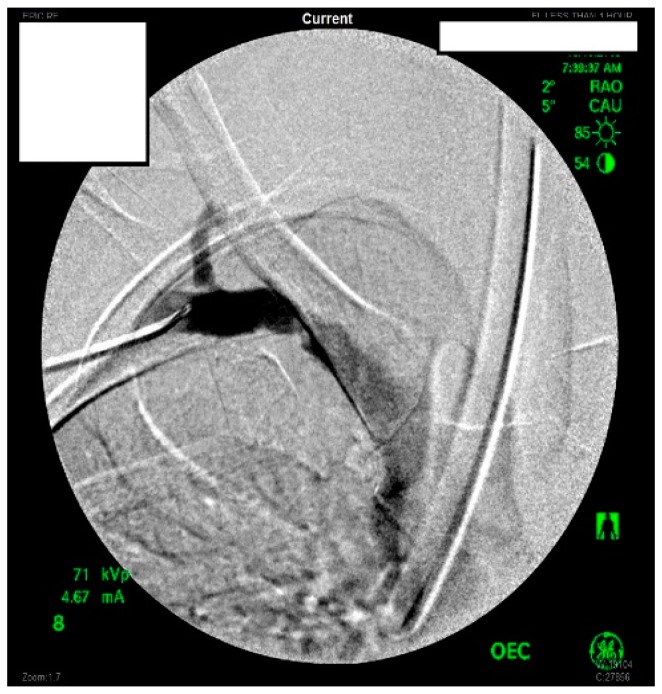
Venogram of a Paget-Schroetter patient in the stress position demonstrating significant stenosis of the right subclavian vein.

**Figure 2:**
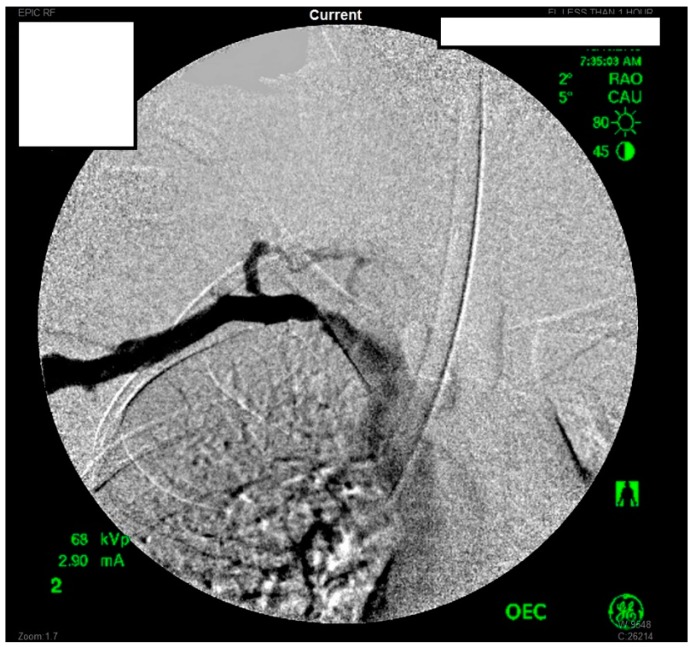
Venogram demonstrating a right subclavian vein that is nearly occluded while in stress position.

**Figure 3:**
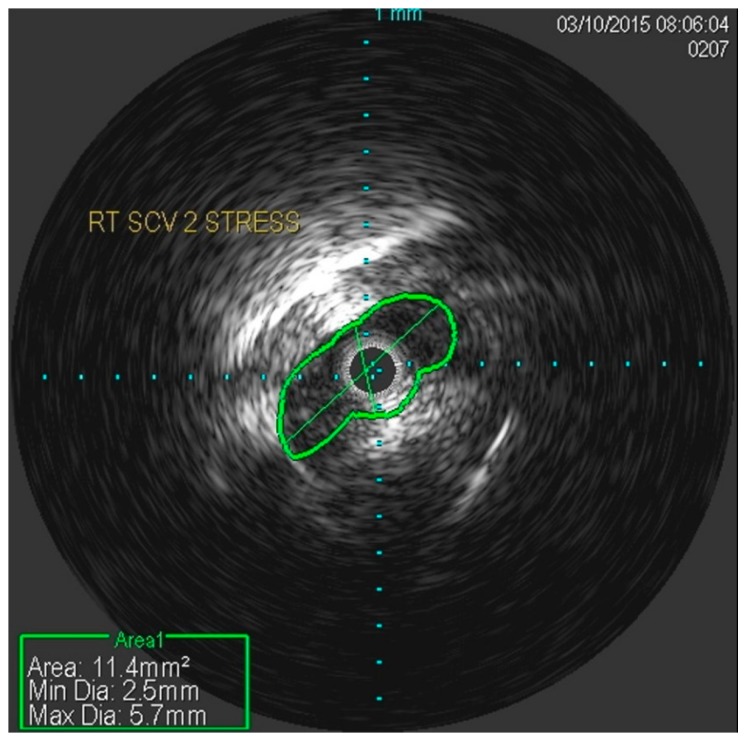
Nearly occluded right subclavian vein in stress position as demonstrated by IVUS.

**Figure 4:**
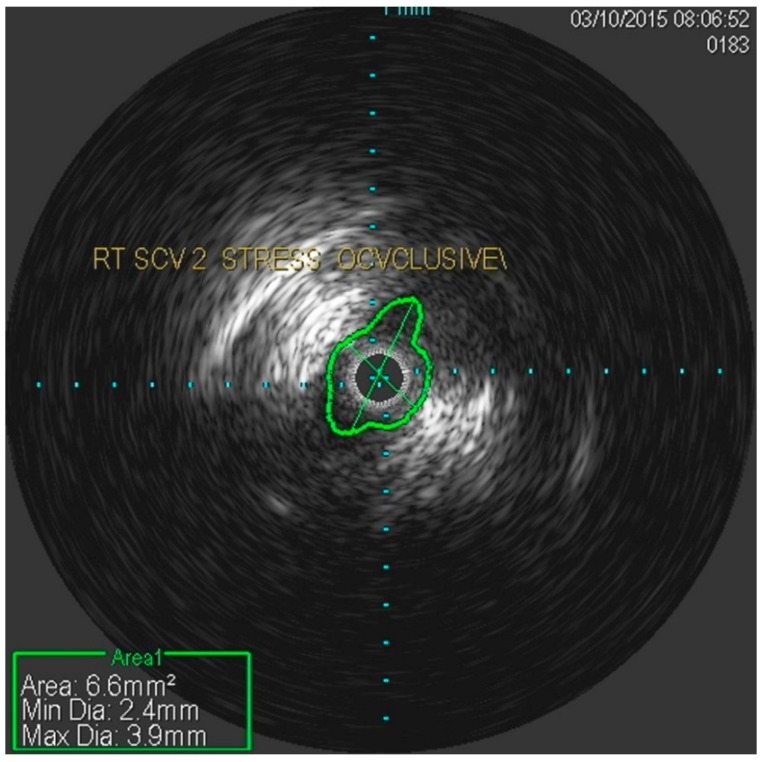
Occlusion of the right subclavian vein in stress position as demonstrated by IVUS.

She underwent venography of the right arm venous system which revealed a 70–80% stenosis of the right subclavian vein in neutral position (Figure 1 and Figure 2). The vein was completely occluded in stress position. This was confirmed with intra-vascular ultrasound (IVUS). On IVUS measurements, the neutral position yielded a 74.4% stenosis while the stress position yielded 100% total occlusion (Figure 3 and Figure 4). It was noted that the contralateral vein appeared compressed in the costochondral space as well, though she was asymptomatic.

She underwent trans-axillary first rib resection. She recovered quickly and was discharged two days post-operatively. She resumed Rivaroxaban on post-operative day 5. She was brought back for a post-operative venogram with IVUS two weeks post-operatively. This revealed a high grade stenosis of the subclavian vein at the thoracic outlet. The lesion was treated with a 12 mm diameter balloon, which was effective.

A left-sided venogram was performed simultaneously, which revealed high-grade stenosis of the subclavian vein at the thoracic outlet. She was brought back two months afterwards for a trans-axillary first rib resection given the significant compression on venography and IVUS. It is important to note that treatment of the contralateral side is not widely accepted. However, we opted to proceed after a thorough discussion with the patient regarding her alternatives. She was kept on anticoagulation one month post-operatively, then discontinued. She was followed for up to one year post-operatively with no recurrence of symptoms and full resolution of normal activity.

## 8. Diagnosis of Arterial Thoracic Outlet Syndrome

Arterial thoracic outlet syndrome (ATOS) is a rare phenomenon typically seen in young, healthy individuals. It is frequently a result of a bony anomaly leading to subclavian artery compression and repetitive trauma. This results in arterial changes including aneurysmal dilatation, stenosis, or ulceration. The anatomical changes that typically cause ATOS include the cervical rib, anomaly of the first rib, or bony spurs that result from previous bone fractures [19,20]. The incidence of ATOS in TOS patients is approximately 6% [5].

Clinical presentation of ATOS is similar to arterial sufficiency in any extremity. The spectrum of symptoms ranges from effort fatigue to subacute or acute limb ischemia, which occur in approximately 50% of patients [21]. Rarely, posterior stroke symptoms may occur (approximately 5% of patients). If significant aneurysmal degeneration has occurred, a pulsatile mass in the shoulder or upper chest may be described (15% of patients) [21]. History typically includes repetitive overhead activity, and subtle physical exam findings may include splinter hemorrhages distally.

Our experience indicates that a subclavian artery duplex is a valuable initial study once the diagnosis of ATOS is suspected. Duplex ultrasonography allows for the visualization of the artery and provides vital information including the size of the artery, flow characteristics, presence or absence of thrombosis, and distal perfusion. We then obtain a CTA of the affected extremity to assist in operative planning; however, this study is sometimes omitted if the duplex has provided this information. Our gold standard for diagnosis is catheter-based angiography, although modern MR and CT angiography allows for avoidance of catheter-based procedures unless there is also an intention to treat. Essential findings include aneurysmal or ulcerative degeneration, size of lesion, mural thrombus, and distal emboli [5,17].

## 9. Management of Arterial Thoracic Outlet Syndrome

Management of ATOS is dictated by the presentation. Acute arm ischemia in these patients is treated first. If the ischemia is not limb-threatening, thrombolysis is performed, especially if there is evidence of thrombosis in the digital arteries as these are difficult to treat surgically. Thrombolysis follows the same principles outlined above. Access is achieved by femoral or radial approach depending on arch anatomy. Our preferred method is to with a 4F micropuncture needle, then upsize to a short 5F sheath. A catheter is placed within the target vessel, and Alteplase (Genentech) is infused at a rate of 0.5 mg/kg/h while heparin runs through the access sheath at 400 units/hr. Serial PTTs and fibrinogen levels are followed. Lysis checks are performed under angiography every 24 h while strict neurovascular checks are performed every two hours in the intensive care unit.

If the patient presents with acute limb-threatening ischemia, these patients are emergently taken to the operating room for exploration and open thrombectomy. The operative approach depends on the site of arterial occlusion. This can be determined by a combination of physical examination and noninvasive/radiographic findings. Emboli frequently lodge at branch points, and it is common to have a cutoff in the brachial artery. If imaging suggests a limited embolus, a simple cut-down and embolectomy at the brachial artery can be performed. Retrograde approaches to clot removal can also be performed from a brachial approach. Using careful technique, clot can also be removed from the radial and ulnar arteries via a brachial approach. On-table angiography is then performed; if residual thrombus is seen, simultaneous thrombolysis can be performed. There are occasions where consideration must be given to upper extremity fasciotomies, so the limb should be carefully evaluated following revascularization.

Once the upper extremity is revascularized, we continue the patient on therapeutic anticoagulation while a definitive plan is made for decompression and arterial reconstruction. Bony anomalies are the typical etiology in ATOS—more so than the venous or neurogenic subtypes. For these issues, a first rib resection or resection of the bony prominence is essential. The presence of a cervical rib can be as high as 75% of patients. In these patients, we prefer to resect both the first and cervical ribs by supraclavicular approach. In approximately 12% of patients, a first rib abnormality alone is the issue; in these patients, we opt for a transaxillary first rib resection [21]. Repairing the subclavian artery is performed at a later stage by supraclavicular approach. Other bony prominences such as a healed clavicular fracture occur in less than 10% of patients. In these circumstances, we perform a first rib resection in addition to resection of the bony prominence [21].

## 10. Approach to Subclavian Artery Repair in ATOS

The supraclavicular approach provides excellent exposure to all anatomic structures associated with the thoracic outlet. It is useful in repairing the subclavian artery in ATOS patients and provides the possibility of simultaneous decompression, though we prefer a staged approach in order to ensure a complete first rib resection.

With the patient in supine position and a transverse roll placed beneath the shoulders, the sternal notch, clavicle, and sternocleidomastoid muscle are identified. A transverse incision is made 1 cm superior to the clavicle just lateral to the sternocleidomastoid. Dissection through the subcutaneous tissue and platysma is performed. The external jugular will be encountered and should be ligated. The sternocleidomastoid is then divided. This should be performed carefully, as the carotid sheath lies directly beneath. This dissection should be carried out carefully on the left side to avoid injuring the thoracic duct as well, which drains at the confluence of the internal jugular and subclavian veins.

The scalene fat pad is then dissected along the medial border and reflected laterally to expose the anterior scalene muscle. The phrenic nerve is carefully identified, and the scalene muscle transected. Once transected, the subclavian artery will be easily visualized [22]. Once exposed, the artery may be repaired with a biologic interposition graft, direct primary repair, or ligation and distal bypass. These options have similar patency rates as well as patient functionality outcomes [21].

In terms of endovascular approaches, there is a paucity for data for the use of covered stents in the subclavian artery in ATOS. Historically, subclavian artery stents have a low 1-year patency rate, as low as 60% when used for aneurysm repair [23]. We have occasionally utilized this technique for ATOS patients. In one case, a young, athletic patient with a subclavian artery aneurysm from ATOS was treated with a covered stent (Viabahn, Gore, Flagstaff, AZ, USA), which remained patent for 9 years while on aspirin. The stent occluded and he presented with acute arm ischemia, which was successfully treated with thrombolysis. The secondary patency period is yet to be determined. In a different case, a young woman was also treated with an endograft (Viabahn), which remained patent for two years. She then returned with recurrent claudication and significant in-stent restenosis requiring angioplasty with a drug-eluting balloon. Though we have not investigated this data closely, it is likely that long-term patency of this technique may be inferior to traditional reconstruction.

## 11. Contralateral Asymptomatic Lesions

As in VTOS patients, a subgroup present with unilateral symptoms while having marked compression of the contralateral side on imaging. We favor treating these patients with decompression prophylactically, though we realize this is disputed. Transaxillary first rib resection is our preferred method in these patients as well.

## 12. ATOS Case Presentation 

**Figure 5:**
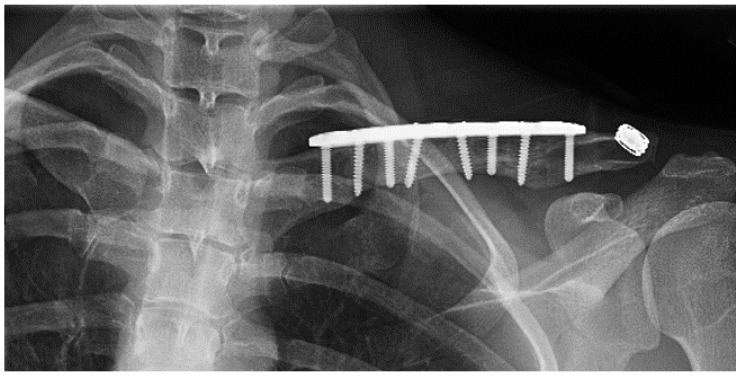
Arterial thoracic outlet syndrome (ATOS) patient with metal plate and screws after a prior clavicular fracture—the screws are abutting the thoracic outlet.

**Figure 6:**
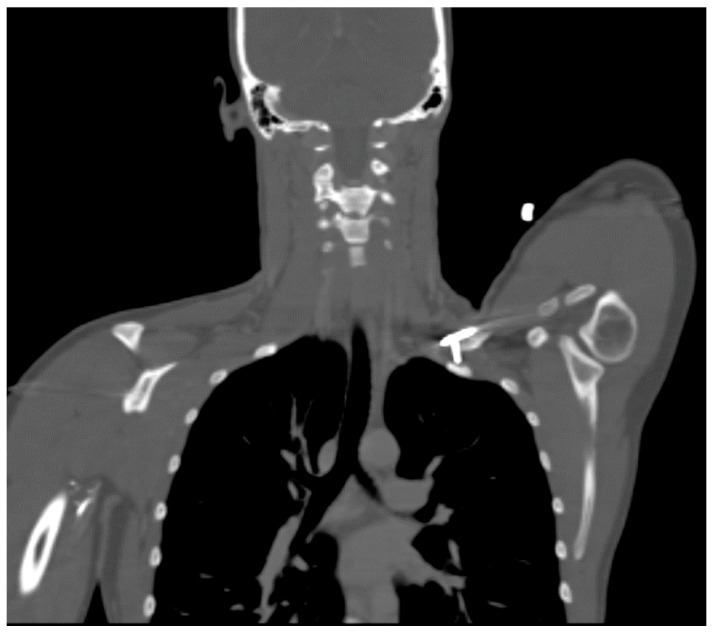
CT angiogram demonstrating a screw abutting the thoracic outlet in an ATOS patient with a subclavian artery aneurysm.

A 27 year-old female who had suffered a left clavicular fracture that was repaired previously presented with left upper extremity numbness and pain for one week. The symptoms occurred spontaneously and were intermittent throughout the week. She experienced no relief with analgesics. She presented to our Emergency Department. Brachial, ulnar and radial pulses were non-palpable. An arterial duplex revealed an occlusive thrombus of the brachial artery at the mid-humerus that appeared to be associated with the patient’s previous clavicular repair (Figure 5). A CT angiogram subsequently revealed a subclavian artery aneurysm adjacent to one of the screws from her prior clavicle repair (Figure 6).

**Figure 7:**
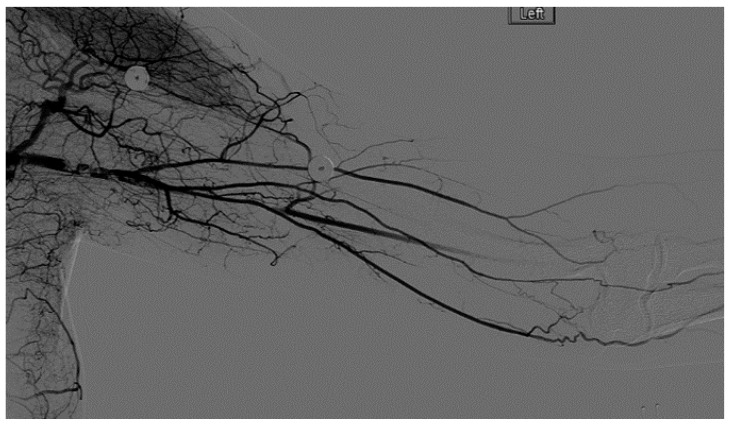
Left subclavian arteriography demonstrating a thrombosed brachial artery at the mid-humeral level and extensive collateralization proximally.

**Figure 8:**
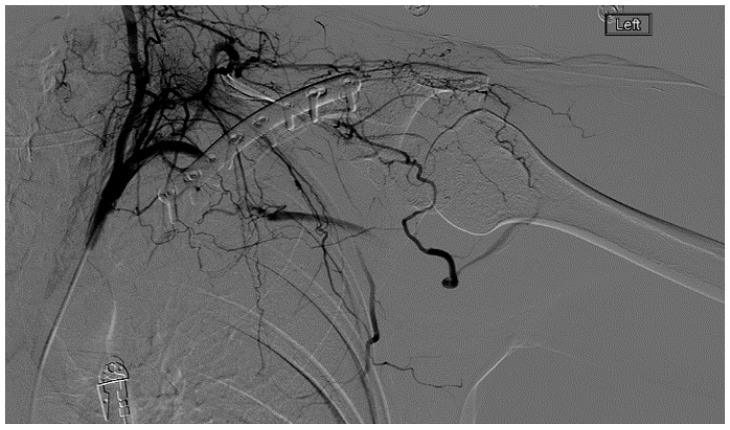
Left subclavian arteriography in an ATOS patient in stress position demonstrating a totally occluded subclavian artery.

**Figure 9:**
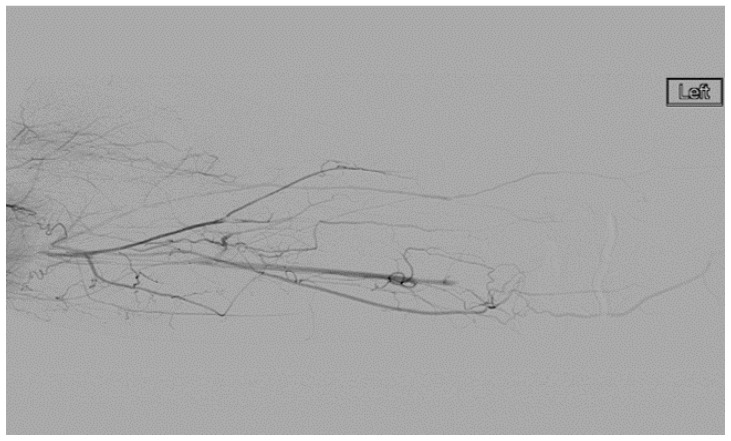
Distal left arm angiography in an ATOS patient demonstrating an occluded radial artery at the origin and ulnar artery at the mid-forearm.

**Figure 10:**
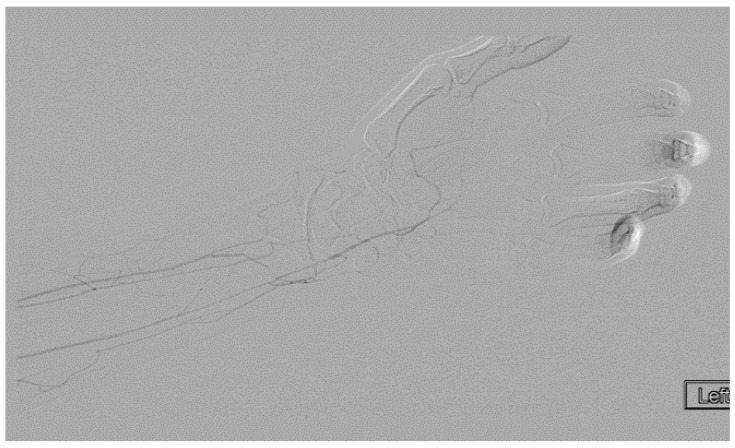
Angiography of the radial and ulnar arteries as well as the palmar arch and digital branches in an ATOS patient with a brachial thrombus.

**Figure 11:**
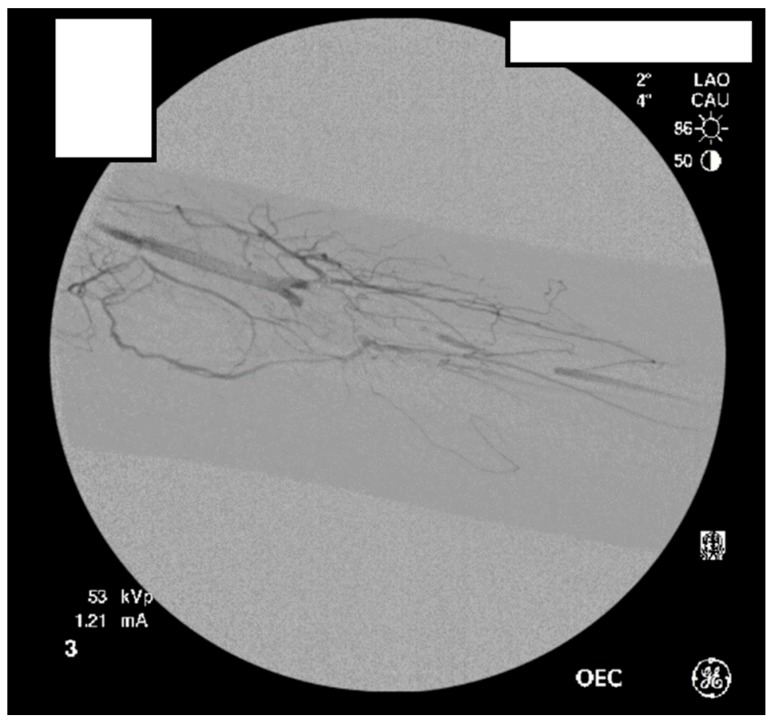
Distal brachial artery thrombosis in an ATOS patient.

**Figure 12:**
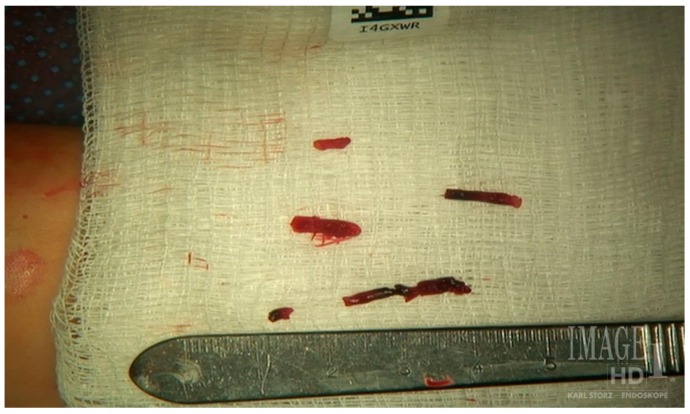
Excised thrombus from the brachial and radial arteries of an ATOS patient with a subclavian artery aneurysm.

A heparin drip was then initiated, and she was taken to the catheterization lab for thrombolysis. This was carried out with Alteplase for 48 h as the patient had strong collaterals and was not in limb-threat (Figure 7, Figure 8, Figure 9, Figure 10 and Figure 11). However, her radial artery remained occluded. She was then taken to the operating room for thromboembolectomy of the left brachial and radial arteries (Figure 12). She was continued on anticoagulation post-operatively and was discharged.

Two months later, she was brought in for a trans-axillary left first rib resection and a placement of a 7 mm by 5 cm Viabahn endograft to exclude the subclavian aneurysm. Anticoagulation was withheld 3 days prior to the operation. Orthopedic Surgery was consulted to remove the adjacent screws simultaneously. She was discharged on Aspirin and Plavix two days post-operatively.

She was followed every six weeks for 18 weeks, then every 3 months the first year. She received arterial duplex ultrasounds at the 3-month, 6-month, and 12-month intervals for the first year. These revealed mild in-stent restenosis, which was stable.

She presented nearly two years later with recurrent left arm claudication. An angiogram revealed a significant in-stent restenosis that was significantly flow-limiting. This was treated with a 6 mm × 4 cm paclitaxel-coated balloon. Her symptoms improved, and she has been followed up to over 18 months after the secondary intervention without symptoms. A discussion was held as to whether treatment of the contralateral side was warranted. We did not proceed with treatment, as she was asymptomatic and compression of the right side was not demonstrated to the same degree as the left. As mentioned earlier, treatment of contralateral limbs is not widely accepted and should only be done after a thorough discussion with the patient regarding the efficacy of further intervention.

## 13. Conclusions

**Figure A1:**
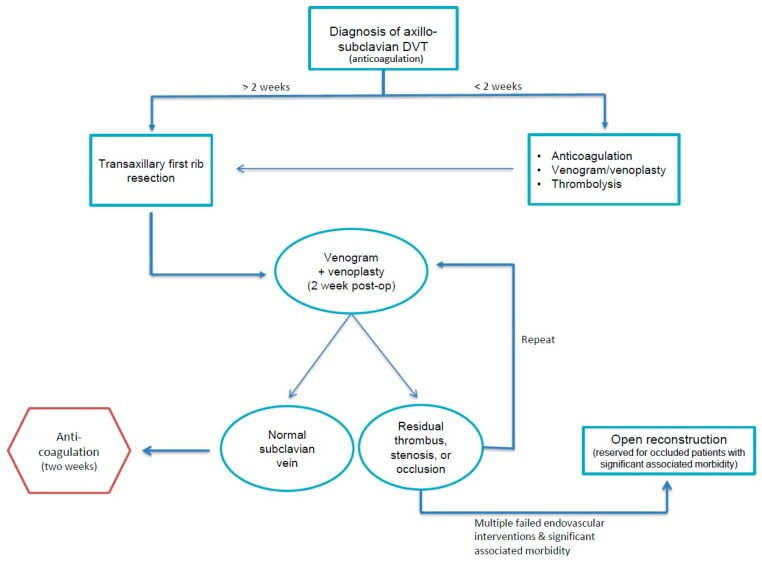
VTOS.

Vascular thoracic outlet syndrome is a rare disorder. The principles of managing venous TOS revolve around decompression and keeping the subclavian vein patent, if possible. These include early diagnosis, swift thrombolysis, and transaxillary decompression. More importantly, it is essential to commit to an efficacious algorithm such as the one outlined in this paper. Work up typically begins with duplex ultrasound and is followed by venography and thrombolysis or a first rib resection depending on the findings. This is followed by a two-week post-operative venogram and anticoagulation for one month. Patients with persistent symptoms are treated with repeated efforts of thrombolysis (Figure A1).

**Figure A2:**
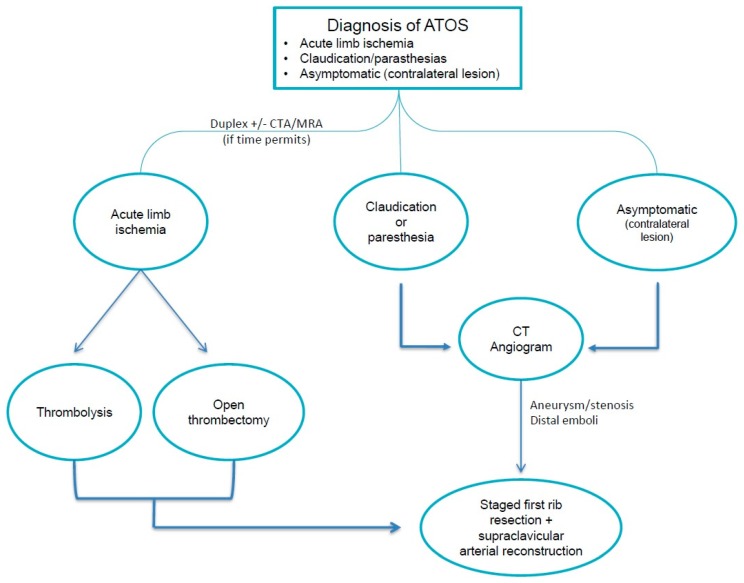
ATOS.

Managing arterial TOS depends on the presentation of the patient. The degree of ischemia may dictate a rapid, open approach to the arterial compromise, although many patients can safely undergo thrombolysis. Lytic therapy is much more likely to allow for complete resolution of the distal thrombus that can occur in these patients. As with venous TOS, definitive therapy requires decompression of the thoracic outlet and ultimately, repair of the injured vessel (Figure A2).
